# The Burden of Asymptomatic Malaria Infection in Children in Sub-Saharan Africa: A Systematic Review and Meta-Analysis Exploring Barriers to Elimination and Prevention

**DOI:** 10.1007/s44197-025-00365-2

**Published:** 2025-02-05

**Authors:** Daniel Asmelash, Wubetu Agegnehu, Wondaya Fenta, Yemane Asmelash, Shibihon Debebe, Abyot Asres

**Affiliations:** 1 Department of Medical Laboratory Science, College of Medicine and Health Sciences, Mizan- Tepi University, P.O Box 260, Mizan-Aman, Ethiopia; 2 School of Public Health, College of Medicine and Health Sciences, Mizan-Tepi University, Mizan-Aman, Ethiopia; 3 Department of Statistics, Bahirdar University, Bahirdar, Ethiopia; 4 Department of Statistics, Aksum University, Aksum, Ethiopia; 5 Department of Medical Laboratory Science, Bahirdar Health Science College, Bahirdar, Ethiopia

**Keywords:** Asymptomatic malaria, Children, Prevalence, Sub-Saharan africa, Systematic review and Meta-analysis

## Abstract

**Background:**

Malaria remains a major public health problem that continues to cause death in under-five children nearly every minute. The purpose of this systematic review and meta-analysis was to determine the pooled prevalence and predictors of asymptomatic malaria in children in Sub-Saharan Africa.

**Methods:**

Relevant studies were retrieved from Web of Science, Cochrane Library, PubMed, Google Scholar, Gray Literature, Embase, and African Online Journal databases published between 2014 and 2024. Data quality was assessed by a tool developed by Hoy and colleagues and classified as low, moderate, or high risk of bias. We performed a random effects model and sub-group analysis by age group, region, and diagnostic methods. The protocol was registered in the PROSPERO (CRD42024584354).

**Results:**

A total of 24 cross-sectional studies with 19,169 participants from 10 Sub-Saharan Africa countries were included in the analyses under the age of 15 years. The overall prevalence of asymptomatic malaria was 25% (95% CI: 20–30%) and showed no evidence of publication bias. Utilization of insecticide-treated nets was significantly associated with asymptomatic malaria. In addition, the overall prevalence of anemia in asymptomatic *Plasmodium*-infected children under the age of 15 was found to be 35% (95% CI: 24–46%). Subgroup analysis showed significant regional and diagnostic tool differences in asymptomatic P*lasmodium* infection.

**Conclusion:**

The findings of this study revealed a high prevalence of asymptomatic *plasmodium* infection in children with significant regional variations. There was a significant association with anemia and the utilization of insecticide-treated nets.

**Supplementary Information:**

The online version contains supplementary material available at 10.1007/s44197-025-00365-2.

## Introduction

Malaria remains a major global health problem that continues to cause death in under-five children nearly every minute. This preventable and highly curable disease is disproportionately concentrated in Sub-Saharan Africa (SSA), which bears the burden most [1]. Malaria is still the biggest threat to the health of the people in Africa, especially in the SSA region which constitutes the largest proportion [2].

According to the Global Malaria Report 2022 statistics, 123 countries and territories reported malaria cases; there were 249 million confirmed malaria cases and 608,000 malaria deaths on aggregate. In the World Health Organization African Region, 233 million people were reported to be infected with *Plasmodium* parasites, accounting for 95% of global malaria deaths, with children under five accounting for 80% of malaria deaths [3]. Sub-Saharan African countries accounted for the majority of these cases and deaths and under-five children were the most affected demographic, representing 76% of the deaths [1].

Most countries in SSA have made considerable progress in household ownership of at least one insecticide-treated nets (ITNs) in recent years, with an average coverage of 67% in 2022, compared to 14% in 2005 [1, 4]. Insecticide-treated nets have played a crucial role in decreasing transmission and mortality rates following the distribution of nearly 2.5 billion ones since 2000 [1, 4]​.

Despite the impressive gains that have been made in malaria control and prevention in the past two decades millions of children are still vulnerable. Among these children, asymptomatic malaria is one of the biggest threats. Unlike symptomatic malaria, asymptomatic malaria does not cause the symptoms of fever and chills, so it is usually not diagnosed and treated, allowing the parasite to live and multiply in a community [5]. Asymptomatic malaria in children who have no symptoms is of immense epidemiological significance because such individuals are silent carriers of the disease making eradication efforts difficult. Their role in sustaining the transmission cycle, even in areas with strong control measures, makes understanding and addressing asymptomatic infections crucial [5, 6].

Malaria infections in children are often asymptomatic, contributing to community transmission as ‘hidden’ parasite reservoirs with respect to endemic regions, intensity of malaria transmission and age of the children [5, 7]. The prevalence of asymptomatic malaria in children is high, with significant rates of co-infection with other diseases such as helminths, which complicates the clinical picture and management. For example, the symptoms associated with helminth infection include fatigue, abdominal pain, and anemia, making it difficult to attribute specific symptoms solely to malaria or helminths. Asymptomatic malaria in children under 15 is associated with an increased risk of anemia, which can have long-term health impacts [8, 9].

It is important to determine the magnitude of asymptomatic malaria, as well as factors that may determine infections in children, it is therefore critical for the development of effective intervention strategies and policies for malaria eradication [10]. The proportion of asymptomatic malaria varies widely in children depending on geographic, socio-economic, and genetic factors, as well as the factors that may determine infection in children [10]. Major host factors encompass age, immune status, nutritional status, and genetic makeup through which the host becomes susceptible to asymptomatic *Plasmodium* infection. The following aspects also relate to parasite-related factors, including species, and genetic differences. Environmental factors such as vector density, seasonality, climate, and the distance to water sources significantly influence malaria transmission [10–12].

We conducted this systematic review and meta-analysis to systematically pool data on the burden of and factors associated with asymptomatic malaria in children in SSA. The findings will help inform public health strategies and policies to reduce the hidden burden of asymptomatic malaria and therefore achieve the goal of malaria elimination in Africa.

### Objectives

- To estimate the pooled prevalence of asymptomatic malaria infection in children in SSA countries.
- To estimate the pooled magnitude of anemia among asymptomatic malaria infected children in SSA countries.
- To identify and analyze predictors associated with asymptomatic malaria infection in children in SSA.
- To assess variations in prevalence and predictors across different regions, age groups, and diagnostic methods.

## Methods

### Search Strategy

To retrieve relevant research, the following electronic bibliographic databases were searched: Web of Science, Cochrane Library, PubMed, Google Scholar, Gray Literature, Embase and regional databases (Africa-wide Information, AJOL) from August 5, 2024 up to August 29, 2024. To ensure thoroughness and avoid duplication, we searched for existing systematic reviews and meta-analyses on this topic using the PROSPERO database (http://www.library.ucsf.edu/). The protocol was registered in the PROSPERO number (CRD42024584354). The keywords and search terms used included: “Malaria,” “Asymptomatic Malaria,” “Prevalence,” “Magnitude,” “Risk Factors,” “Predictors,” “Determinants,” “Africa,” “Child,” “Children” and the names of specific countries within SSA.

### Eligibility

The inclusion criteria for this systematic review and meta-analysis were designed to ensure the selection of relevant and high-quality studies. The pediatric population of interest was defined as children aged 6 months up to 15 years in SSA countries, representing the affected demographic for asymptomatic malaria. Asymptomatic malaria is the condition of interest, where the disease being examined is malaria at infection without clinical symptoms to target those people who are infected but do not exhibit symptoms. Included study designs will be cross-sectional and cohort studies that provide substantial data on the prevalence and forms of asymptomatic malaria and its predictors. Furthermore, studies must report on prevalence rates or identify predictors associated with asymptomatic malaria to ensure the data is relevant for calculating pooled prevalence estimates and analyzing significant contributing factors.

Studies focusing on populations outside SSA or on adults, studies not addressing asymptomatic malaria, non-peer-reviewed articles, non-primary research, editorials, commentaries, case reports, reviews, and studies with insufficient data were excluded to ensure that all included studies contributed meaningful and analyzable data to the review.

### Study Selection and Data Extraction

All studies retrieved through database searches and manual searching, deemed potentially relevant for inclusion, were managed using EndNote X9. Initially, duplicate studies were excluded. Subsequently, the titles and abstracts of the remaining studies were screened to identify those that met the inclusion criteria. Titles and abstracts that did not meet the inclusion criteria were excluded at this stage. Following reduplication and preliminary screening, potentially relevant studies were further evaluated by reviewing their full text. Studies that did not meet the pre-specified criteria were excluded during this phase.

Data were extracted from eligible studies using a prepared Excel sheet. The extracted data included author(s) and year of publication, country of study, region, age group, diagnostic methods used, hemoglobin status, sample size, number of children tested, and number of asymptomatic malaria-positive participants. The process of study selection and data extraction was carried out independently by four authors (DA, YA, WF & AA), and any inconsistencies or disagreements were resolved through consensus. Factors included in this study were anemia status, ITN utilization, stunting, and family infection history.

### Quality Assessment

The quality of the included studies was accurately evaluated by three authors independently using the risk of bias assessment tool developed by Hoy et al. [13], specifically designed for cross-sectional studies. This tool assesses both internal validity (data collection, case definition, study instrument reliability and validity, data collection method, prevalence period, and correctness of numerator and denominator) and external validity (representation, sampling frame, random selection, and non-response bias). Each study was assessed according to ten items and a score of one (yes) or zero (no) was assigned for each item. Overall quality ratings were categorized as low (> 8), moderate (6–8), or high (≤ 5) risk of bias based on the overall score. Additionally, systematic reviews and meta-analyses were evaluated for adherence to the PRISMA guidelines to ensure transparency and reproducibility.

### Statistical Analysis

All statistical analyses for this study were performed using the Metan command in the Stata software V.11. To synthesize the pooled odds ratios (ORs), data were extracted from 2 × 2 tables, with a continuity correction of 0.5 added in cases where there were zero events. Crude and adjusted odds ratios and 95% CI were determined to assess the association between the asymptomatic malaria infection and anemia, ITN use, stunting, age group, and family (sibling) infection.

The pooled prevalence and adjusted odds ratio (AOR) across the studies were computed specifically using the DerSimonian-Laird method in a random-effects model. The I² statistic was used to assess heterogeneity among the studies.

Publication bias was checked through visual inspection of funnel plots, and it was more formally assessed using Egger’s regression test and Begg’s correlation test. Diagnostic methods, region, age group and risk of bias were used to perform subgroup analyses to identify potential sources of heterogeneity. Additionally, a univariate meta-regression analysis was performed based on the publication years of the studies and sample sizes. Moreover, to test the robustness of the overall pooled estimates, a sensitivity analysis was conducted by systematically excluding one study at a time.

## Result

### Study Selection

**Fig. 1:**
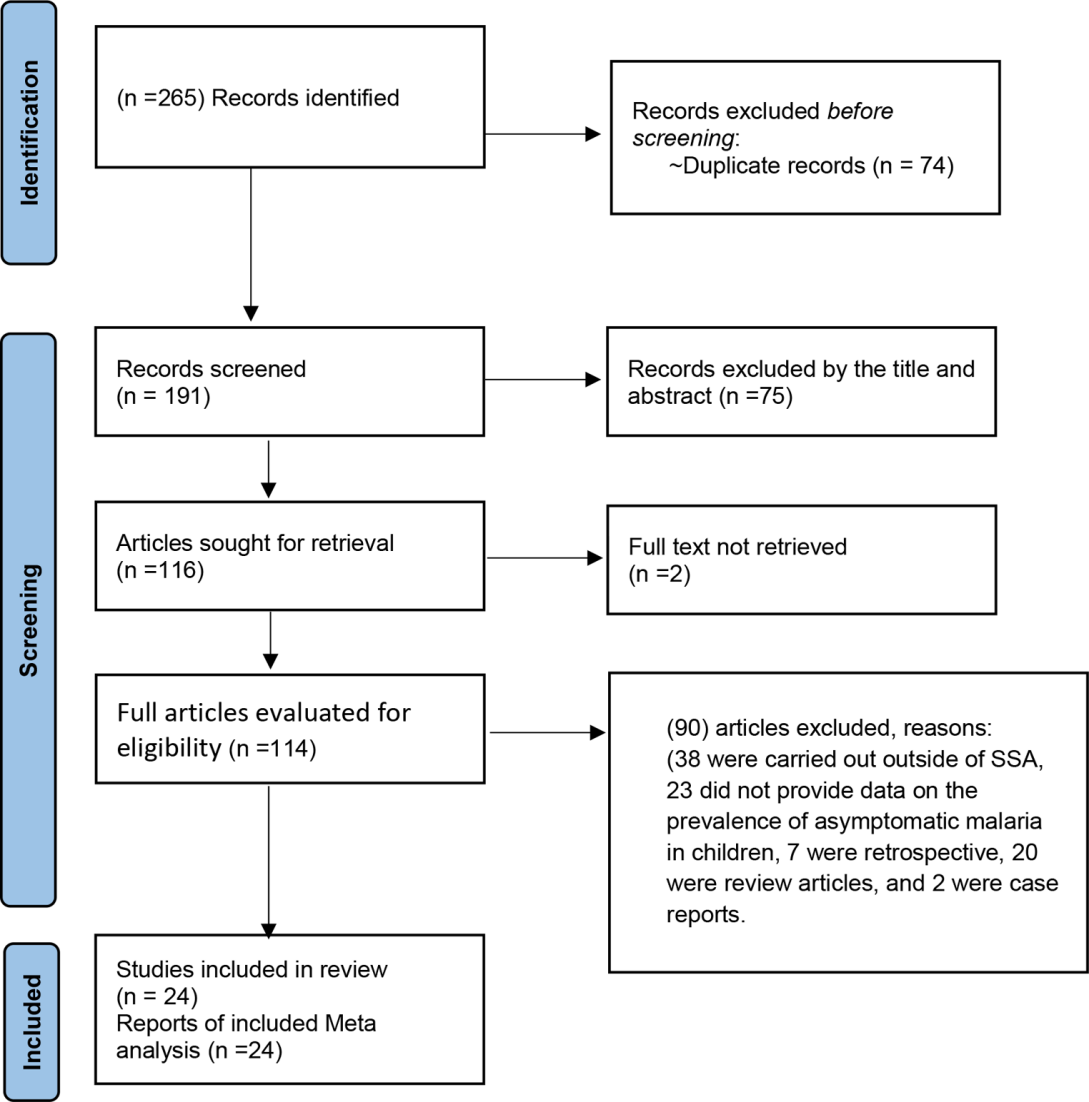
PRISMA 2020 flow diagram of the searched, identified and included studies of prevalence and predictors of asymptomatic malaria in Children in SSA

From the database search, we had a total of 265 records; after the removal of the duplicates, we were left with 191 records for initial screening based on their titles and abstracts. We excluded 75 records by title and abstract and three of those were not in English. A total of 114 studies were then subjected to review at the full text level. From this group, 90 articles were excluded for the following reasons: 38 were conducted in countries other than SSA, 23 had no report on the asymptomatic prevalence, 20 were review articles, 7 were retrospectives, and 2 were case reports. Therefore, 24 papers were considered in the final meta-analysis (Fig. 1).

### Characteristics of Included Studies

The characteristics of 24 eligible studies were included in this systematic review and meta-analysis. The studies were performed in ten countries over the past ten years, from 2014 to 2024. A total number of 19,169 study participants were included in this study, with sample size ranging from 127 to 4,892. Four of the included studies were done in Ethiopia, three in Tanzania, DRC, and Ghana, two in Kenya, Uganda, Nigeria, and Cameroon respectively, and one each in Malawi, Rwanda, and the Central African Republic. Nine studies out of the included studies diagnosed asymptomatic malaria through microscopy only, four through rapid diagnostic tests (RDT), six through RDT, microscopy, and PCR, four through RDT and microscopy, and one through PCR only. For the risk of bias assessment, 15 of the studies seemed to have a low risk of bias, while 9 studies had a moderate risk of bias (Table 1).

**Table 1:** Selected characteristics and quality assessment of the studies included in the systematic review and meta-analysis(*n* = 24)

No	Author, year	Country	Study design	Diagnostic method	Age group	Sample size	Asymptomatic Malaria Prevaence	Quality Assessment Hoy et al.,2012[13]
1	Ntenda PAM et al., 2022[14]	Malawi	Cross-sectional	RDT	5-15year	684	25.8	Low
2	Wudneh F et al., 2021[15]	Ethiopia	Cross-sectional	Microscopy	5-15year	413	11.1	Moderate
3	Zerdo Z. et al., 2021[16]	Ethiopia	Cross-sectional	RDT	5-15year	2167	1.6	Low
4	Sumari D.et. al., 2016[12]	Tanzania	Cross-sectional	Combined (RDT, Mic &PCR)	5-15year	501	22.0	Low
5	Akindeh NM et al., 2021[17]	Cameroon	Cross-sectional	Combined (RDT, Mic &PCR)	5-15year	127	66.9	Moderate
6	Okoyo C et al., 2021[18]	Kenya	Cross-sectional	Combined (RDT, Mic &PCR)	Under 5	542	34.1	Low
7	Maziarz M.et. al., 2018[19]	Uganda	Cross-sectional	RDT and Microscopy	0-15year	942	11.8	Low
8	Peprah S. et al., 2019[20]	Kenya	Cross-sectional	RDT and Microscopy	0-15year	965	29.1	Low
9	Lufungulo BY et al., 2020[21]	DRC	Cross-sectional	Combined (RDT, Mic &PCR)	Under 5	1,088	24.8	Low
10	Sumbele IUN et al., 2021[22]	Cameroon	Cross-sectional	Microscopy	5-15year	1271	34.0	Low
11	Akiyama T et al., 2016[23]	DRC	Cross-sectional	PCR	0-15year	319	6.3	Moderate
12	Nundu SSet. al., 2021[24]	DRC	Cross-sectional	Combined (RDT, Mic &PCR)	5-15year	427	45.7	Low
13	Biruksew A et al., 2023[7]	Ethiopia	Cross-sectional	Combined (RDT, Mic &PCR)	5-15year	994	2.9	Low
14	Orish V et al., 2018[25]	Ghana	Cross-sectional	RDT and Microscopy	5-15year	550	50.4	Low
15	Mensah BA et al., 2021[6]	Ghana	Cross-sectional	Microscopy	5-15year	4892	4.3	Low
16	Worku L et al., 2014[26]	Ethiopia	Cross-sectional	Microscopy	5-15year	385	6.8	Moderate
17	Nzobo BJ et al., 2015[11]	Tanzania	Cross-sectional	Microscopy	5-15year	317	5.4	Moderate
18	Hayuma PM et al., 2021[27]	Tanzania	Cross-sectional	RDT and Microscopy	5-15year	565	25.8	Low
19	Agaba BB et al., 2022[28]	Uganda	Cross-sectional	RDT and Microscopy	0-15year	288	34.7	Moderate
20	Korzeniewski K. et al., 2021[29]	Central Republic	Cross-sectional	RDT	0-15year	500	15.2	Low
21	Nyirakanani C et al., 2018[30]	Rwanda	Cross-sectional	Microscopy	Under 5	222	12.2	Moderate
22	Kanwugu ON et al., 2019[31]	Ghana	Cross-sectional	Microscopy	0-15year	345	2.6	Moderate
23	Ajayi IO. et al., 2015[32]	Nigeria	Cross-sectional	Microscopy	5-15year	365	52.3	Moderate
24	Abah AE, et al., 2015[33]	Nigeria	Cross-sectional	Microscopy	5-15year	300	63.3	Moderate

### Publication Bias Assessment

Hence, from the funnel plot symmetry which was done qualitatively, there appears to be no evidence of publication bias (Supp 1 Fig). To quantitatively confirm the funnel plot results, we performed both Egger’s regression test and Begg’s correlation test. Egger’s regression test (*P* = 0.562) and Begg’s correlation test (*P* = 0.286) both indicated the absence of publication bias. (Supp 1 Table).

Table 1 Prevalence of Asymptomatic Malaria Infection in SSA Countries, 2024.

**Fig. 2:**
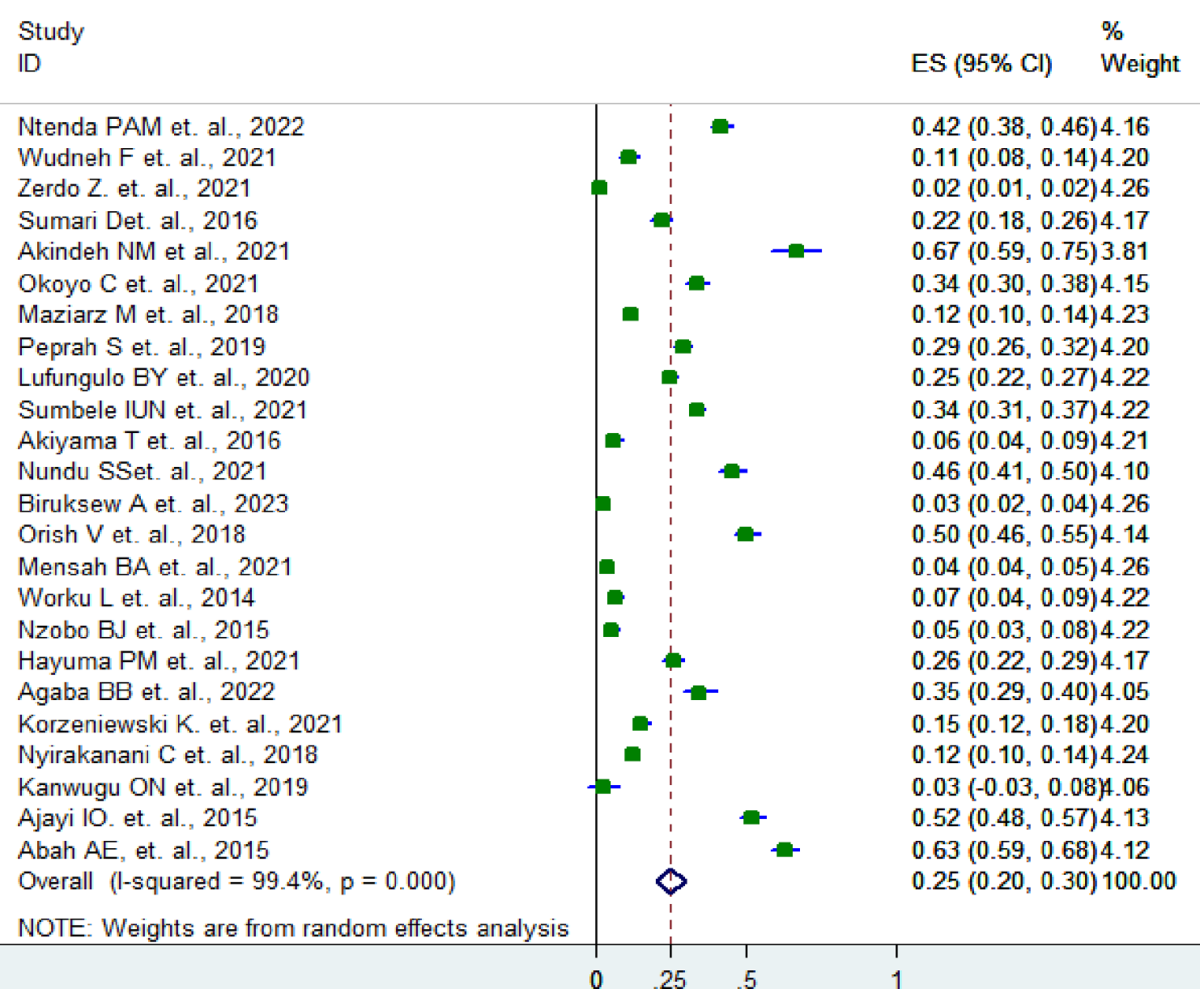
Forest plot of asymptomatic malaria infection among children in the SSA countries, 2024

The pooled prevalence of asymptomatic malaria infection in SSA children was found to be 25% (95% CI: 20–30%) using a random-effects model (Fig. 2).

### Subgroup Analysis

**Fig. 3:**
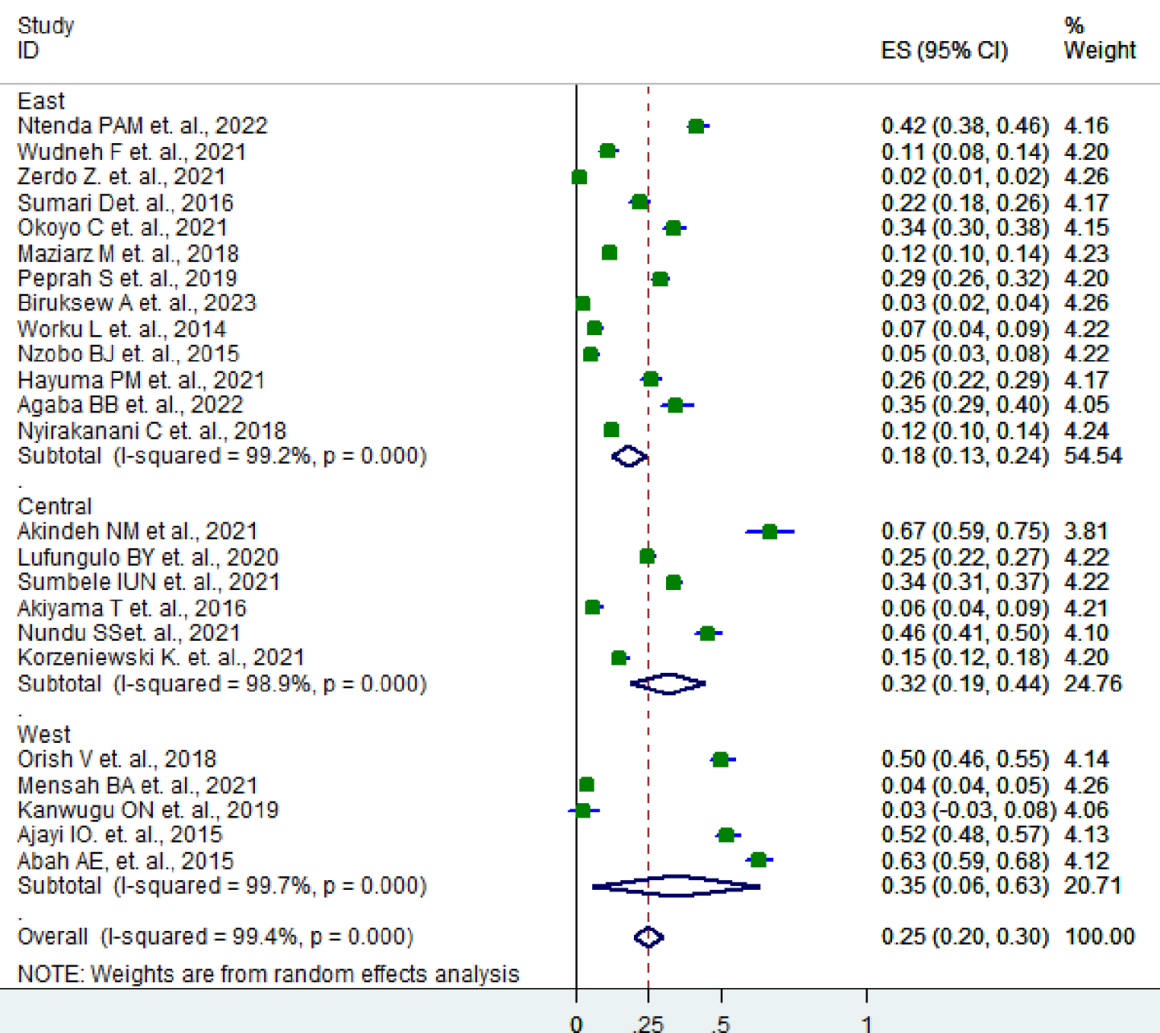
Subgroup analysis of asymptomatic malaria infection according to the region in which the SSA countries are located, 2024

The subgroup analysis based on the region of the studies revealed that asymptomatic malaria infection in children was more prevalent in the West African countries 35% (95% CI: 6–63)) followed by central Africa 32% (95% CI: 19–44) and twice more prevalent than in East African countries 18% (95% CI: 13– 24) (*P* < 0.001) (Fig. 3).

**Fig. 4:**
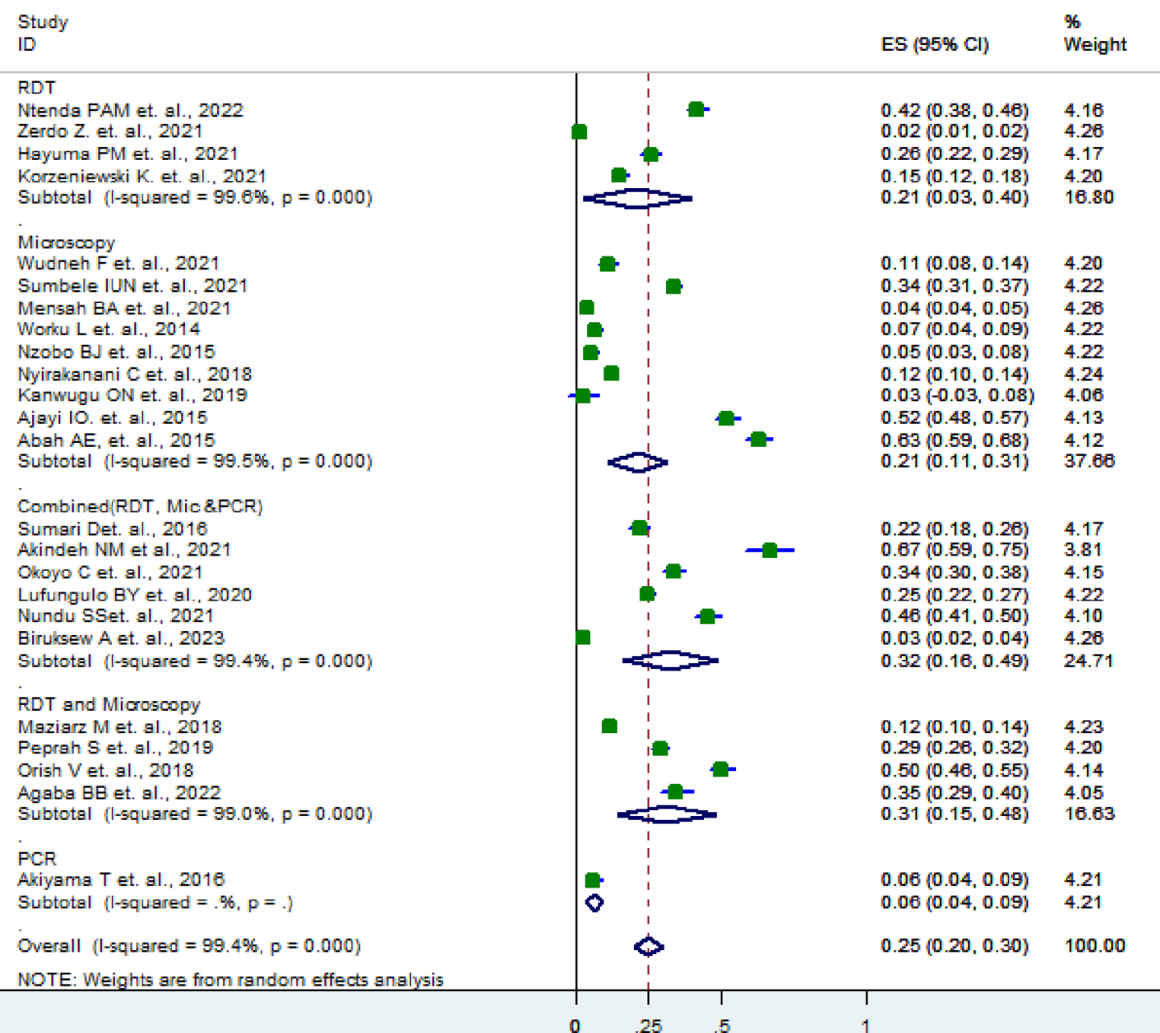
Subgroup analysis of asymptomatic malaria infection by the test methods in the SSA countries, 2024

The overall pooled estimate of asymptomatic malaria infection was significantly higher in studies that diagnosed asymptomatic malaria using a combination of PCR, RDT and Microscopy 32% (95% CI: 16–49), and a combination of microscopy and RDT methods 31% (95% CI: 15–48), compared to studies that used microscopy 21% (95% CI: 11–31%) and rapid diagnostic methods alone 21% (95% CI: 3–40) (Fig. 4).

In addition, the pooled estimate of asymptomatic *plasmodium* infection was significantly higher in studies among the 5–15 age group 29%, (95% CI: 22–35) compared to studies that were conducted among under five children (17%, 95% CI: 8–25) (Supp 4 Fig.).

**Fig. 5:**
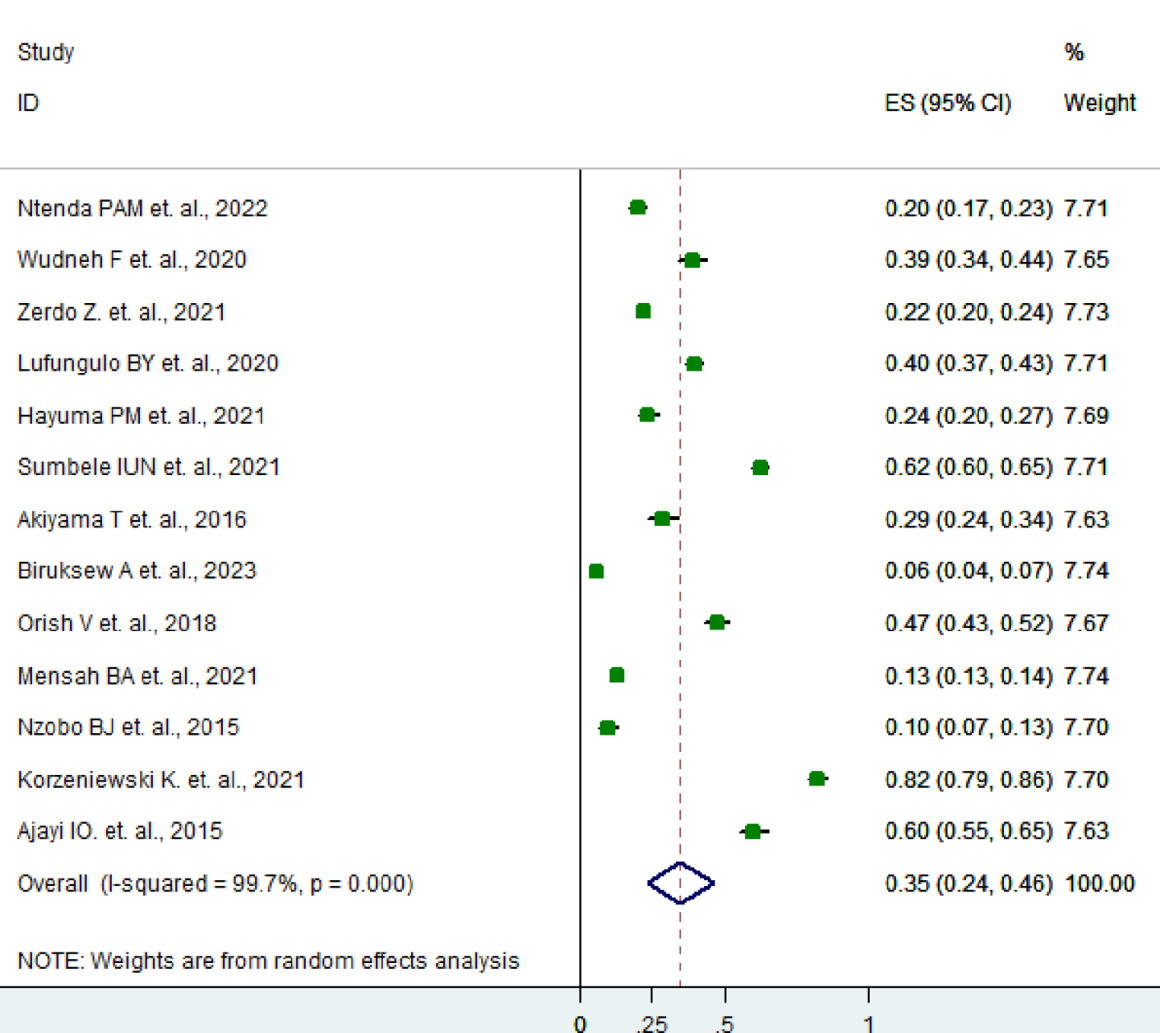
Prevalence of anemia among asymptomatic malaria infected children in the SSA, 2024

Moreover, out of the twenty-four included studies, 13 reported anemia prevalence among asymptomatic malaria infection. The overall pooled prevalence of anemia in asymptomatic malaria infected children was found to be 35% (95%, CI: 24–46%) using a random-effects model (I² = 99.7%, *P* < 0.001) (Fig. 5).

### Meta-Regression and Sensitivity Analysis

**Fig. 6:**
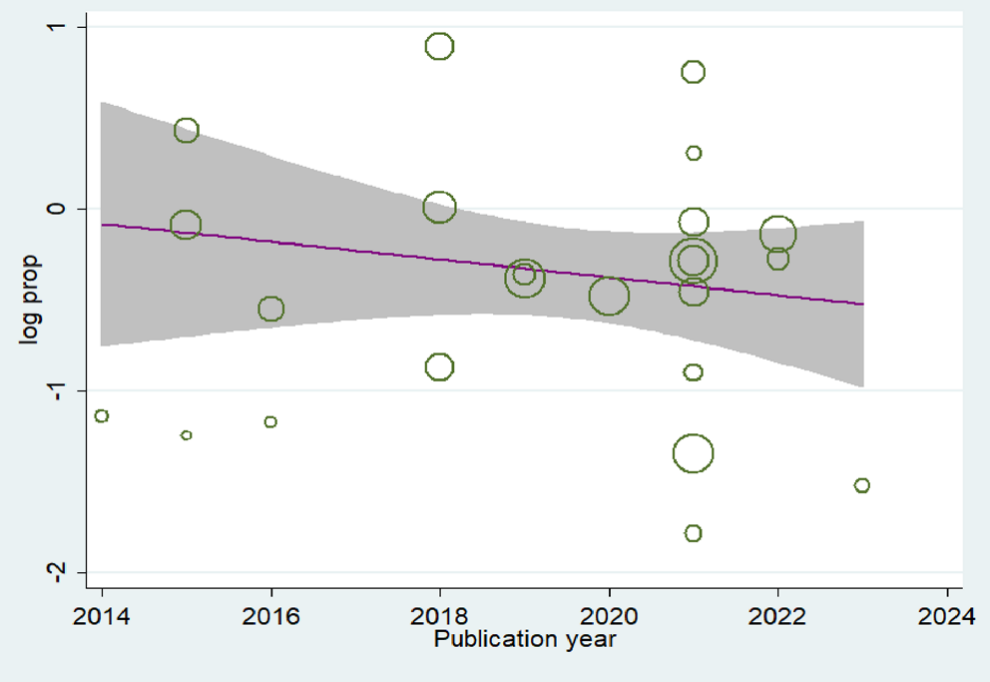
Meta-regression plot showing the trend of prevalence of asymptomatic malaria infection in children in SSA over a ten-year period

We performed the meta-regression analysis to assess the trends of asymptomatic malaria infection, and it indicated that the prevalence of asymptomatic malaria infection did not decrease significantly during the years 2014 and 2024 (Coef. -0.039, *P* = 0.944) (Fig. 6).

The findings of the present sensitivity analysis revealed that the overall pooled estimate was robust and not influenced by any single study. When individual studies were omitted one at a time, the highest overall prevalence estimate was 26.05% (95% CI: 20.12–31.95%) and the lowest was 23.22%, (95% CI: 18.57–27.88%) which was very close to the pooled overall prevalence estimate of 25%, (95% CI: 21–31.2%). This consistency showed the stability and reliability of the pooled prevalence estimate (S2Fig). Furthermore, the meta-regression of the trends of anemia showed that the prevalence of anemia did not decrease significantly (Coef. -0.1586, *P* = 0.636) (Fig S.3).

### Association between Anemia and Asymptomatic Malaria Infection

**Fig. 7:**
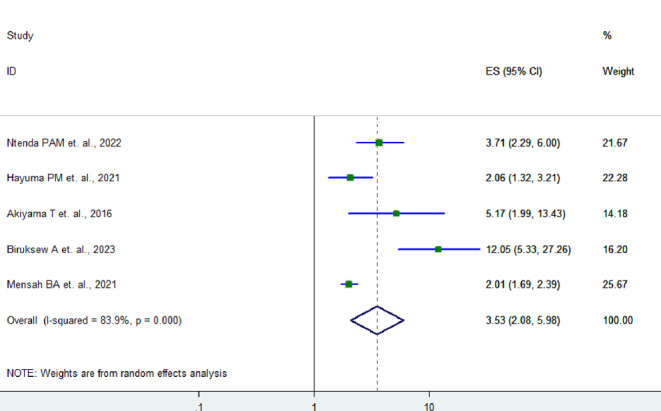
Forest plot showing the association between anemia and asymptomatic malaria infection in children in SSA, 2024

Five studies showed that asymptomatic malaria infected children had higher odds of anemia than non-infected children by 3.53 times (AOR = 3.53, 95% CI: 2.06–5.98) (Fig. 7).

### Association between ITN Utilization and Asymptomatic Malaria Infection

**Fig. 8:**
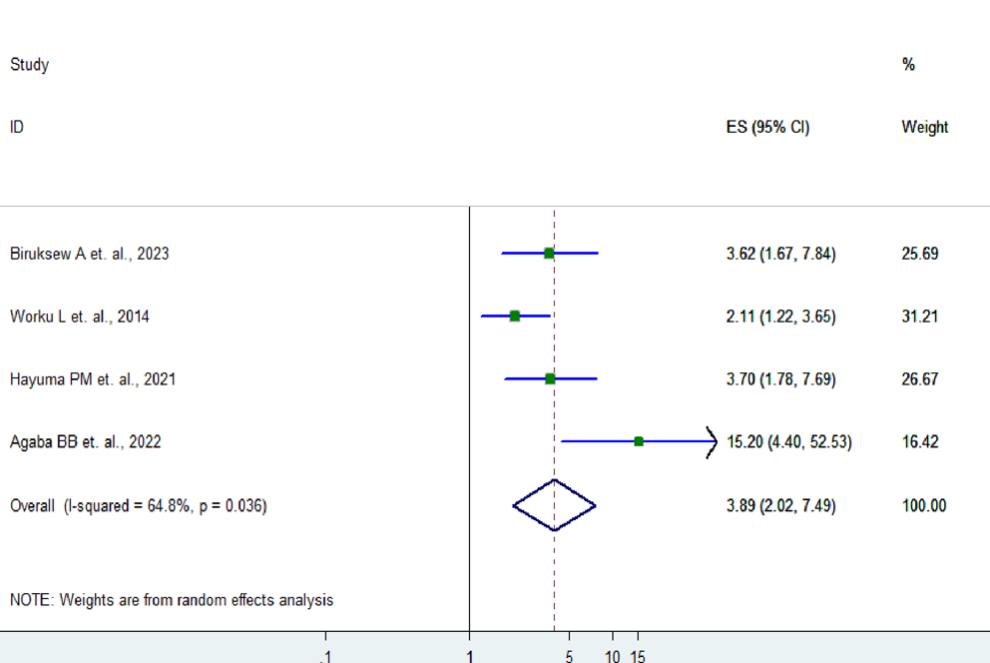
Forest plot showing the association between ITN utilization and asymptomatic malaria infection in children in SSA, 2024

Families who never or sometimes used ITN were 3.89 times more likely to have asymptomatic malaria compared to families who usually utilized ITN (AOR = 3.89, 95% CI: 2.02–7.49) (Fig. 8). However, the analysis showed that there was no significant association between asymptomatic malaria and stunted children and family infection history.

## Discussion

Asymptomatic malaria constitutes a major problem for malaria control and elimination efforts in SSA. Individuals with asymptomatic malaria do not exhibit overt clinical symptoms; therefore, they are not likely to seek health care and sustain the transmission of malaria. These findings clearly showed the need for targeted strategies to address this hidden burden, especially among children, who are often overlooked in malaria control programs.

In the present systematic review and meta-analysis, we included the cross-sectional studies that reported an overall prevalence and factors associated with asymptomatic malaria in children in SSA. All the included studies involved ten SSA countries with diverse geographic distributions, and the different diagnostic methods used in the studies increased the external validity of our findings. This systematic review and meta-analysis aimed to synthesize existing evidence on the prevalence and determinants of asymptomatic malaria in children in SSA.

Our meta-analysis findings showed that the overall prevalence of asymptomatic malaria in children is 25% (95% CI: 20–30%). This relatively high prevalence indicates a huge malaria burden among those who are asymptomatic in the region, which in turn poses a challenge to malaria elimination in the region.

This finding confirms the worrying level of asymptomatic malaria in the region, which was much higher than the point prevalence of asymptomatic *Plasmodium* infection in enrolled participants in Asia [34], (Southeast Asia, South Asia, and Western Asia were 5.8%, 9.4%, and 8.4%, respectively), Eastern India 8.4% [35] and central India 13.6% [36].It was comparable to pooled estimates in pregnant women (26.1%) [37]. However, it was lower than that of Sweden (35.8%) [38] and Bangladesh (30.7%) [39]. The high prevalence observed in moderate to high transmission areas may be due to difference in classification criteria or diagnostic methods, the frequency of reinfection with *Plasmodium* and the development of partial anti-malaria immunity.

Subgroup analysis showed that the prevalence rates differed depending on the diagnostic methods used. The molecular method showed the highest overall prevalence of 32% (95% CI: 16–49), compared to microscopy 21% (95% CI: 11–31%) and RDT methods alone 21% (95% CI: 3–40). Such differences can be explained by the fact that molecular testing possesses higher sensitivity, permitting the detection of very low levels of parasitemia that might not be identified using microscopy and RDTs. This finding is supported by a meta-analysis of the diagnostic accuracy of microscopy and RDTs, which exhibit very low sensitivities [40, 41]. This finding suggests the need to identify more accurate tools for detecting asymptomatic infection, such as PCR-based tests, to be used in malaria control and elimination strategies.

Our findings showed a higher prevalence of asymptomatic malaria in the 5–15 age group (29%, 95% CI: 22–35) compared with children under five (17%, 95% CI: 8–25). This difference could be attributed to the fact that in endemic areas, there can be higher asymptomatic infection rates in older children as they are more likely to carry the parasite without displaying symptoms. This highlights the potential role of older children as hidden reservoirs for malaria transmission, emphasizing the need for targeted interventions within this age group. The subgroup analysis showed regional differences in the prevalence of asymptomatic malaria. Children in West Africa exhibited the highest prevalence at 35% (95% CI: 6–63%), followed by Central Africa at 32% (95% CI: 19–44%) and East Africa at 18% (95% CI: 13–24%). These differences could be due to regional variations in malaria transmission, the vectors, and the extent of control measures [42]. West and Central Africa had high cases, which probably means that these areas need more intervention to manage the asymptomatic carriers of malaria.

The overall pooled prevalence of anemia in asymptomatic children infected with malaria was found to be 35% (95% CI: 24–46%). It is understood that anemia is a recognized complication associated with malaria, and our results presented here have lent stronger evidence to support the relationship between asymptomatic malaria and anemia in pediatric populations. The effects of asymptomatic malaria in children with anemia are that the condition deteriorates and hampers their health greatly.

Moreover, asymptomatic malaria infected children were 3.53 times more likely to be anemic compared to their non-infected counterparts (AOR = 3.53, 95% CI: 2.06–5.98). This association is comparable with the study done in Lao, Japan, where asymptomatic malaria was found to be associated with anemia [OR 5.17, 95% CI 1.99–13.43] [23]. The pathophysiology of malaria anemia is not fully understood and is multifactorial, which can involve both hemolysis and deficient erythropoiesis [43, 44]. This strong association needs a coordinated health system approach to managing both malaria and anemia to optimize the positive development of child health.

Our study also showed that families who never or sometimes used ITNs were 3.89 times more likely to have asymptomatic malaria infection compared to families who used ITNs consistently (AOR = 3.89, 95% CI: 2.02–7.49). This finding is supported by the pooled estimate of the study conducted among pregnant women in Ethiopia 6.93; (95% CI: 3.27–14.71) [45]. This present study confirm the importance of ITNs in controlling malaria transmission and eliminating asymptomatic malaria. This indicates that enhancing the level of ITN coverage and pursuing consistent utilization can be useful approaches to drastically reduce the reservoir of asymptomatic malaria.

However, the current study revealed that the prevalence of asymptomatic malaria was not significantly associated with that in non-stunted children. In contrast, a study conducted in Lao, Japan showed that stunted children were more likely to be infected with asymptomatic malaria 3.34, (95% CI: 1.25–8.93) [23].

Despite these findings, this study had several limitations. The literature search was limited to English-language studies, potentially introducing language bias. A high level of heterogeneity was observed among the studies, likely due to differences in diagnostic methods and publication years. This study is crucial to understanding the magnitude of asymptomatic malaria in children from SSA and underscores the importance of using accurate diagnostic techniques as well as specific disease control efforts. Future research should aim to include a broader range of studies across different African countries and explore strategies to address the identified gaps in malaria elimination efforts.

## Conclusion

This study established that SSA children have a high prevalence of asymptomatic malaria infection in children, which has significant regional variations and associations with anemia and the utilization of insecticide-treated nets (ITNs). Additionally, there was a high prevalence of anemia among asymptomatic malaria-infected children. West and Central Africa remain the regions with a significantly higher prevalence of asymptomatic malaria, calling for an urgent need for more targeted and comprehensive intervention measures in these parts of the region.

The significant association between the utilization of ITNs and a lower prevalence of asymptomatic malaria revealed the essential need to advocate for and secure the widespread and regular use of ITNs as a key malaria control strategy. Moreover, the connection between asymptomatic malaria and anemia in children emphasizes the necessity for integrated health interventions that tackle both malaria and its related complications. Our study offers a crucial understanding of the hidden burden of asymptomatic malaria and its public health implications in SSA. These results underscore the urgent need for improved diagnostic methods, focused interventions, and ongoing initiatives to reduce the burden of asymptomatic malaria, ultimately aiding in the wider objectives of malaria control and eradication in the region.

## Electronic Supplementary Material

Below is the link to the electronic supplementary material.

Supplementary Material 1

## Data Availability

No datasets were generated or analysed during the current study.

## Abbreviations

AOR: Adjusted odds ratio
I2: Cochrane heterogeneity index
CI: Confidence interval
OR: Odds ratio
ITNs: Insecticide-treated nets
PCR: Polymerase chain reaction
PRISMA: Preferred Reporting Items for Systematic Reviews and Meta-Analyses
RDTs: Rapid diagnostic test
SSA: Sub-Saharan Africa
WHO: World health organization
