# Supplementary material for: The Burden of Asymptomatic Malaria Infection in Children in Sub-Saharan Africa: A Systematic Review and Meta-Analysis Exploring Barriers to Elimination and Prevention
Source: J Epidemiol Glob Health. 2025 Feb 5;15(1):17. doi: 10.1007/s44197-025-00365-2 (PMC11799456; doi:10.1007/s44197-025-00365-2)
Supplement: Supplementary file 1 — Supplementary Material 1 [file 44197_2025_365_MOESM1_ESM.docx]

**Supplementary Table and figures**

Suppl.1 Table. Beggs and Egger test for publication bias assessment. TIF

**Suppl 1 Fig.** Funnel plot of pooled prevalence of asymptomatic malaria infection by region in the SSA countries, 2024. (TIF)

**Suppl 2 Fig.** Forest plot showing sensitivity analysis. (TIF)

**Suppl 3 Fig .** Meta-regression plot showing the trend of anemia among asymptomatic malaria infected children in SSA over ten years period.

**Suppl 4 Fig.** Subgroup analysis of asymptomatic malaria infection by age group in the SSA countries, 2024.

**Suppl 1 Table**_._ Beggs and Egger test for publication bias assessment for Prevalence of Asymptomatic malaria

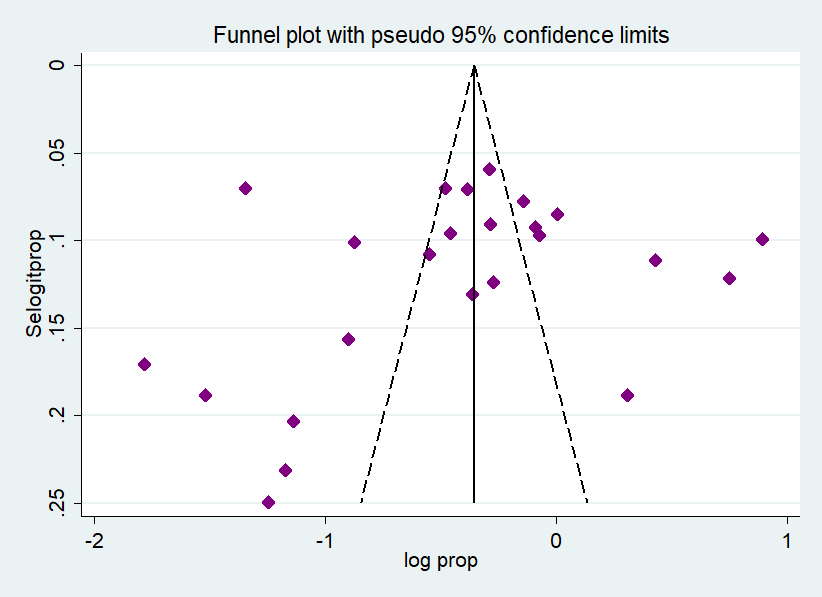


**Suppl 1 Fig.** Funnel plot of pooled prevalence of asymptomatic malaria in children in SSA, 2024.


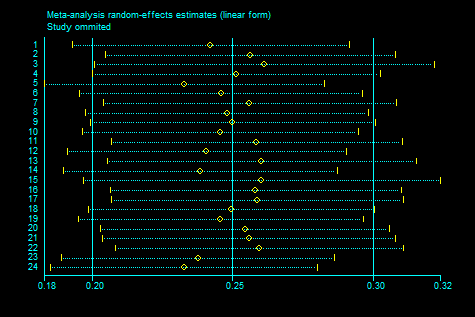


**Suppl 2 Fig.** Sensitivity analysis of pooled prevalence of asymptomatic malaria infection in children

in SSA, 2024


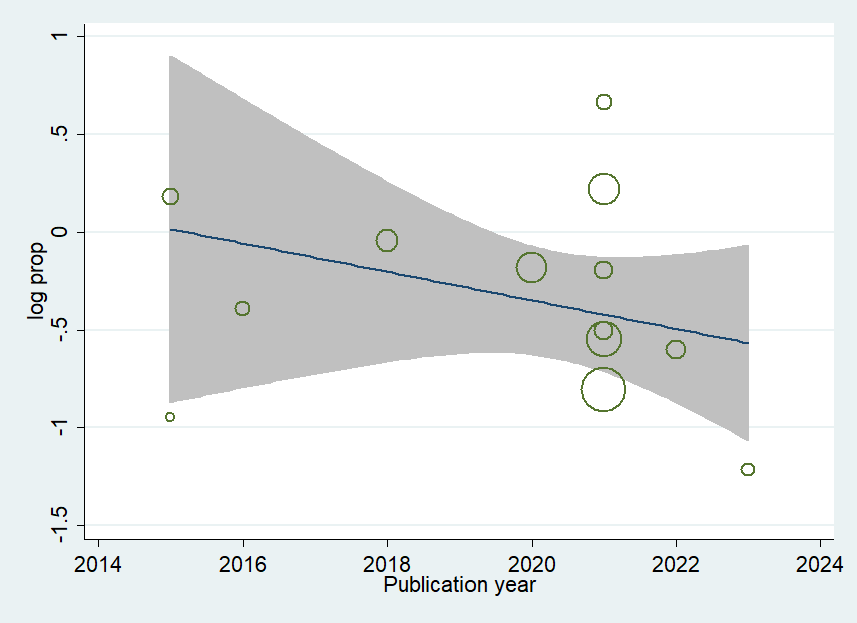


**Suppl 3 Fig3 .** Meta-regression plot showing the trend of anemia among asymptomatic malaria infected children in SSA over ten years period.


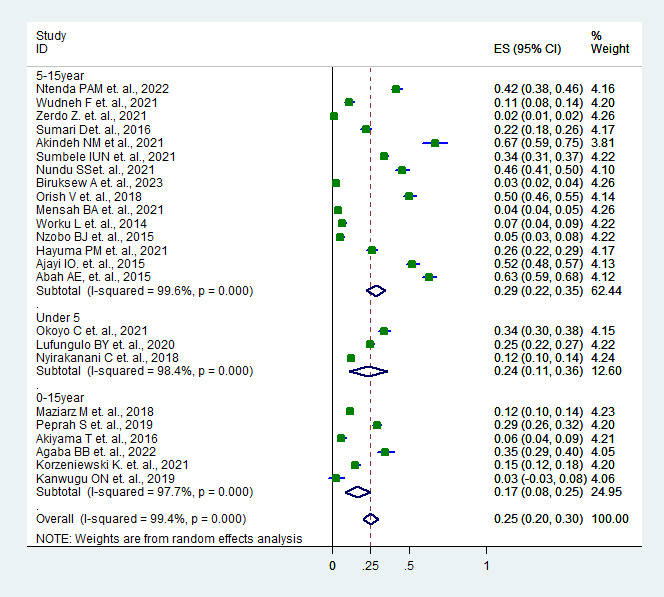


**Suppl 4 Fig.** Subgroup analysis of asymptomatic malaria infection by age group in the SSA countries, 2024.
